# Use of Smokeless Tobacco by Indian Women Aged 18–40 Years during Pregnancy and Reproductive Years

**DOI:** 10.1371/journal.pone.0119814

**Published:** 2015-03-18

**Authors:** Saritha Nair, Jean J. Schensul, Shahina Begum, Mangesh S. Pednekar, Cheryl Oncken, Sameena M. Bilgi, Achhelal R. Pasi, Balaiah Donta

**Affiliations:** 1 National Institute of Medical Statistics, New Delhi, India; 2 Institute for Community Research, Hartford, Connecticut, United States of America; 3 National Institute for Research in Reproductive Health, Mumbai, India; 4 Healis Sekhsaria Institute for Public Health, Mumbai, India; 5 University of Connecticut, Mansfield, Connecticut, United States of America; University of Missouri-Kansas City, UNITED STATES

## Abstract

**Objectives:**

This paper discusses patterns of daily smokeless tobacco (SLT) use and correlates of poly SLT use among married women aged 18–40 years in a Mumbai slum community with implications for tobacco control.

**Methods:**

Using a mixed methods approach, the study included a structured survey with 409 daily SLT users and in-depth interviews with 42 women. Participants for the survey were selected using a systematic sampling procedure (one woman in every fourth eligible household). Univariate and bivariate analysis, and multiple logistic regressions were conducted to identify demographic and social factors associated with women’s use of poly SLT products. To illustrate survey results, in-depth interviews were analyzed using Atlas ti software.

**Results:**

Sixty-four percent of the women surveyed used only one type of SLT; of these, 30% used *mishri*, 32% used *pan* with tobacco and the rest used chewed tobacco (11%), *gul* (17%) or *gutkha* (10%). Thirty-six percent used more than one type of SLT. Poly SLT users chewed or rubbed 50% more tobacco as compared to single users (mean consumption of tobacco per day: 9.54 vs. 6.49 grams; p<0.001). Women were more likely to be poly SLT users if they were illiterate as compared to literate (adjusted odds ratio [AOR]=1.67; 95% confidence interval [CI]=1.07-2.71), if they had lived in Mumbai for 10 years or more, versus less than ten years (AOR=1.67, 95% CI=1.03-2.71); and if their husband was a poly SLT user as compared to a non SLT user (AOR=2.78, 95% CI=1.63-4.76). No differences were noted between pregnant and non-pregnant women in SLT consumption patterns.

**Conclusions:**

Tobacco control policies and programs must focus specifically on both social context and use patterns to address SLT use among women of reproductive age with special attention to poly SLT users, an understudied and vulnerable population.

## Introduction

India is one of the world’s largest producers and consumers of tobacco, much of it in smokeless form (SLT), and available in a variety of different types and brands across the country. Smoking is rare among Indian women, but research over the past decade has shown that Indian women’s use of smokeless tobacco products is substantial [1–3] and increasing, with negative consequences for both oral morbidity [4–8] and perinatal health, including premature delivery, low birth weight and birth length [9–11]. Indian researchers have shown that these consequences are dose responsive, increasing with the amount of SLT used [12]. Though these epidemiologic links are persistent, there is little research that explores the factors driving SLT use during the reproductive years, and especially during pregnancy. Research on this topic is made more urgent by the growing use among low income, poorly educated women of packaged tobaccos and specialized tobaccos with *supari* (betel nut) and flavorings that include chemical toxins and carcinogenic additives [13–15]. These women constitute a substantial market for cheap tobaccos that have high potential for negative impact on both their general and reproductive health [16,17].

Apart from being used, as women claim, to improve oral health and relief from bowel/abdominal problems, women report that smokeless tobacco performs certain valuable functions including companionship through shared use, and stress and tension/*tenshun* reduction. *Tenshun* is derived from the English word “tension”, a term used by women to refer to an emotional state associated with high levels of poverty, low education, heavy labor burden, marital conflict and domestic violence [18,19]. Women also report chewing tobacco to increase available energy for daily workload and heavy labor especially in the face of limited food intake [9] and to suppress hunger. Recognizing that SLT use among women is an increasing problem that needs to be addressed through social/behavioral and policy level approaches [20–22], both Indian and international researchers have called for more research that goes beyond the epidemiology of SLT use and its health consequences to explore patterns of women’s use especially during pregnancy and reproductive years [21,23] and factors contributing to the simultaneous use of multiple forms of smokeless tobacco (poly smokeless tobacco use) during this period.

The concept of “pattern” with respect to tobacco use generally encompasses prevalence, incidence/initiation, frequency of use per week or day, types of tobacco used and whether one or more types of tobacco are used concurrently [24–27]. Other aspects of pattern less frequently mentioned include the ways women apply or chew tobacco, timing of tobacco use during the course of the day in relation to other activities, and poly tobacco use, defined as concurrent use of multiple types of tobacco in a day or other designated period of time [28]. Social exposure to tobacco use is a known contributor to patterns of tobacco use [29–31], but there are few efforts to quantify exposure to types of tobacco in different components of users’ personal networks. Finally, though there is recognition that poly tobacco use (smoking and smokeless tobacco together) constitute a special challenge in the South East Asia Region [21], there is little or no information on the patterns and predictors of Indian women’s poly SLT use. In this paper, we try to address these information gaps to increase understanding of the patterns of SLT use among women that are likely to be important in shaping prevention and treatment policies and programs. We focus on the tobacco products Indian women use, and the quantity and frequency of use of different types of smokeless tobacco products. We discuss how women obtain SLT, build it into their daily lives, use it during their pregnancies and become poly SLT users. Finally, to understand poly SLT use better, we address demographic and contextual predictors of poly SLT use.

## Materials and Methods

### Study Site and Study Population

The paper is based on data collected from a mixed methods study conducted between 2010 and 2013 with married women in a demographically and culturally diverse low income community of Mumbai [28,31,32]. The study site was a designated-slum community of approximately 60,000–70,000 residents and approximately 233,116 m^2^ (23.3 ha) in the southeastern part of Mumbai, situated along a main commercial road. In its history of development and composition, the community is typical of other slum communities across Mumbai, consisting of first and second generation migrants from rural areas of Maharashtra, Uttar Pradesh and other states.

Study inclusion criteria included married women of reproductive age (18 to 40) and daily users of at least one type of smokeless tobacco product. Married women were selected to meet the study goal of understanding patterns of SLT use prior to and during pregnancy. Unmarried women were excluded, since it is uncommon for women in India to become pregnant prior to marriage.

The study included a qualitative and a survey phase.

The survey covered topics including types of tobacco used, history of initiation and use, frequency and amount of use, preferred types and brands of tobacco, purchasing tobacco, reasons for initiation and continuation, use in social network including husband’s use, norms, and patterns of use during pregnancy, lifestyle, tension in relationships, food insecurity, quit attitudes and attempts, and exposure to pro and anti-tobacco information. Amount of tobacco consumed was obtained by demonstration, using local units of use. The survey was pilot tested, translated into Hindi and Marathi and back translated. It was administered to a representative sample of SLT users using systematic random sampling. To obtain the sample, every fourth household was approached and women were screened for eligibility. A total of 1290 households were identified that included women aged 18–40 years (83% of households screened). Of these, a total of 406 households included at least one woman who reported using any form of smokeless tobacco daily for the past seven days. This gave an estimated SLT prevalence rate of 31.4% among women in the study area. A total of 347 women (85.4%) consented and completed the survey. The reasons for refusals as reported by the women were either time constraints or husband/mother-in-law refusal for participation.

To gain a more complete understanding of the patterns of use among pregnant women, a census of all pregnant SLT using women was carried out by identifying pregnant women through house listing, or through key informant introductions to known pregnant women who expressed willingness to be interviewed. Of a total of 223 additional pregnant women, 67 (16.5%) were found eligible for the study, based on study inclusion criteria (age and use of any form of SLT daily for the past seven days) and of these, 62 consented and completed the survey. A comparison of characteristics of pregnant users in the census and women in the systematic random sample indicated no significant differences so they were pooled for analysis, giving a total of 409 SLT users.

During the qualitative phase, researchers identified married women who met the study criteria and explained the study to them. Forty-two women, approximately half of whom were pregnant at the time, agreed to participate in 2 hour in-depth interviews. The in-depth interview, conducted in Hindi or Marathi, covered types of tobacco used, history of initiation and use, frequency and amount of use, preferred types and brands of tobacco, ways of preparing and purchasing tobacco, reasons for initiation and continuation, use in social network including husband’s use, norms, and patterns of use during pregnancy. Completed, transcribed and quality checked interviews were filed and entered into Atlas ti [33] for coding and analysis.

The study was conducted by the National Institute for Research in Reproductive Health (NIRRH), Mumbai, India, and the Institute for Community Research (ICR), U.S.A and was reviewed (NIRRH IRB#D/IECC/117/2009 dated 22 Sept 2009 and ICR IRB #:2009–03 dated Aug 17 2009) and approved annually by both Institutional Review Boards. The NIRRH ethics committee reviewed and approved all the procedures, Participant Information Sheets (PIS) and Informed Consent (IC) forms. To minimize literacy concerns the PIS as well as the IC forms were read aloud to illiterate participants. A copy of the PIS and IC form were also given to respondents so that if they had questions or doubts, they could ask for assistance from family members or others who could read and interpret the information for them. A signature/thumb impression of both literate and illiterate respondents were recorded on the informed consent form and stored at NIRRH. In the case of an illiterate respondent, the procedure took place in front of an independent witness, who then signed the consent form. Exact wording from the NIRRH ethics informed consent form is the following: *“I have read/ have been read out and understood the participation information sheet (of which a copy has been provided to me)*. *The information regarding the purpose of the study*, *safety risks and benefits has been given to me in the language that I understand (Hindi/Marathi)*. *I have had the opportunity to ask queries which have been clarified to my satisfaction*.”

After obtaining written consent, interviewers administered a survey to all respondents in a private setting in their own home or elsewhere in the community. Completed surveys were quality checked for completion and consistency, dates were entered, and surveys were analyzed using SPSS 19.0 software.

### Variables and Measures

For this paper we have drawn upon data obtained from the survey, supplemented with quotes from the coded in-depth interviews extracted to illustrate and help to explain patterns identified in the quantitative data.

#### Qualitative measures

In-depth interviews were grouped into pregnant/non-pregnant women, and single/poly SLT users, using Atlas ti software (Version 5.2). Codes were used to identify and select the components of tobacco use patterns (type, amount, frequency, poly SLT use, duration of use, duration kept in mouth, daily schedule of use, reasons for use, means of obtaining tobacco products, etc.). These codes were used for extracting illustrative quotations and as the basis for determining quantitative pattern variables used in the survey.

#### Demographic variables

Age at the time of the interview, grouped into under and over 30 years of age; education (illiterate/literate); type of family (nuclear or joint); monthly family income (< Rs.5000 or U.S. $81 or less or ≥ Rs.5000 or U.S. $82 or more); duration of stay in Mumbai (above/below 10 years) and current pregnancy status (yes/no).

#### Pattern of use

The pattern of use is measured on the following five dimensions: (a) frequency of use; (b) amount/quantity of use; (c) duration of time SLT product retained in the mouth; (d) timing of first use; (e) means of acquisition of smokeless tobacco. *Frequency of use*: The number of times a specific form of tobacco was used per day was grouped into 1–2 times a day, 3–4 times a day or 5 times or more. *Amount/Quantity of use*: Women were asked on an average how much SLT they used each time, based on typical “units” of application for each type of tobacco (e.g. a tablespoon, a packet, a *pan* (betel quid), or a cupped hand). Packaged SLT users refer to small packets (approximately one chew) and big packets (almost double the size of small packets) and *pan* users refer to the number of *pan* with tobacco used. To evaluate how much tobacco each respondent consumed, amounts were weighed and gram weights of tobacco were calculated for typical units of each type of tobacco (½ tablespoon or small packet, or full packet). For tobacco which is sprinkled on *pan*, five *pan* shop owners provided the amount they sprinkled on a *pan*, and the total weight was calculated on a scale and divided by five, to approximate the amount. Each participant’s total daily gram weight was calculated and used in comparing single and poly SLT users and pregnant and non-pregnant users. Approximately 50% of women used less than 5 grams per day; thus women were divided into those using above or below 5 grams of tobacco per day. *Duration of time SLT product retained in mouth*: Response to the question “How long do you keep this product in your mouth each time you use it”? Response categories were: less than five minutes, 5–10 minutes, 11–15 minutes; 16–20 minutes; more than 20 minutes.

#### Timing of first use

When each type of tobacco was used for the first time during the day, in response to the question: “How soon after you wake up do you usually use (type of tobacco) for the first time”? Response categories for each type of tobacco used were: within five minutes, 6–30 minutes, 31–60 minutes, more than 60 minutes.

#### Means of acquisition of smokeless tobacco

Response to question “Who obtains each type of tobacco for the respondent most of the time”, categorized as the respondent herself, husband, children or others.

#### Exposure to SLT products during childhood

Yes/no responses to whether during childhood the respondent was exposed to family members (father, mother, siblings, relatives, friends and neighbors) who used SLT. Responses were used separately and summed and recoded into a categorical variable “overall exposure” (0 = no exposure; 1 = yes exposure).

#### Husband’s use of SLT products

Respondent’s report of husband’s daily use of 9 different smokeless tobacco types in the past 30 days, recoded into non-user, single user and poly SLT user.

#### Tension

A scale with items reflecting quality of marital relationship composed of 14 items measured with a three point Likert scale (not at all, sometimes, a lot). Examples of items are “My husband is not taking enough care of the children”; “My sexual needs are not met”; “He has another relationship”; “He doesn’t discuss with me”; “He drinks too much alcohol”. Those who reported “not at all” to every item were considered as “no”. A response of “sometimes” or “a lot” to one or more items was considered as “yes”.

#### Exposure to types of SLT products used by social networks

Respondents were asked which of the five types of tobacco (*mishri*, *pan* with tobacco, packaged or loose tobacco, *gul*, *gutkha*) were used by each of the five classes of people close to them (non-spousal household members, relatives living in Mumbai, relatives living outside of Mumbai, friends, and neighbors). Examples of questions were: “Which of these five types of SLT are used by non-spousal household members”; “Which of these five types of SLT are used by relatives in Mumbai” etc. The range of responses within each class was 0 (no type of SLT used) to 5 (all five types of SLT used). The possible range of responses for all five classes was 0 (no type of SLT was used in any group) to 25 (in each group all five types of SLT were used). The index is a count of exposure to different SLTs across the five close associate groupings and is a proxy for social exposure.

#### Food insufficiency

This scale was derived from the Food and Drug Administration (FDA) Food Security Questionnaire Core Module, adapted for the India context [34]. It included 8 items such as “Are there times when you have gone for more than six to eight hours during the day without food because you are busy?” and “Have you ever avoided eating to make sure that others in your family had enough food?”. Those who responded “never” to all eight items were scored as “no”; a response of “somewhat” or a lot” to one or more of the items was scored as “yes”.

#### Type of SLT user

A count of types of smokeless tobacco used during a single day. Women were grouped for analysis into two groups: single users (one type of tobacco only) and poly SLT users (two or more types of tobacco each day).

### Statistical Analyses

Descriptive analyses were conducted to understand the patterns of use of various types of smokeless tobacco products among women. Bivariate analyses and the chi square test were conducted to identify differences in age, education, duration of stay in Mumbai, pregnancy status, type of family, income, childhood exposure to any product, husband’s use of any SLT product, tension, and food insufficiency by type of tobacco users (i.e. single vs poly SLT users). In bivariate analysis with categorical and continuous variables, χ2 test and t test were applied respectively. Multiple logistic regression was performed to calculate adjusted odds ratios (AOR) and 95% confidence intervals (CIs) for the above mentioned independent variables on dependent variable (single/poly SLT use). All analyses were conducted with SPSS version 19.0.

## Results

### Demographics

The mean and standard deviation for age of women interviewed was 29.6± 6.2 years. Twenty-five percent of women surveyed were pregnant at the time of interview. More than half of the women were illiterate. The majority of the women (70.5%) reported Uttar Pradesh as their native place but mean duration of stay in Mumbai was 14.3± 10 years and only 5% of the women had migrated to Mumbai in the past year. Religious affiliation was approximately 50% Muslim and 50% Hindu. Most households consisted of nuclear families (74.1%). Median family income was Rs 6000 (~ U.S. $98) per month, just over the urban poverty line set by the Tendulkar Commission in 2013 of Rs.5000 (~U.S. $81) per household [35]. The mean number of children was 3.32± 1.8. Approximately 16% of women reported earning cash income mainly through home-based income generating activities.

### Patterns of Tobacco Use

Women reported current use of five main types of smokeless tobacco: *pan* (betel quid) with tobacco, *mishri (*toasted powdered tobacco), *gutkha (*packaged tobacco with chemicals and flavoring), chewed (loose and packaged) tobacco and *gul* (powdered tobacco similar to *mishri*, used in northern states). Nearly half of the study sample overall, including poly SLT users, reported using *pan* with tobacco (48%). Those who used m*ishri*, chewed tobacco with various additives (*mawa*, *zarda* etc.) and used *gul* were each approximately 27% of the sample; at the time of the study, 18% reported using *gutkha*. More than one-third of women (36.2%) reported using two or more of the above-mentioned products in different combinations (Table 1).

**Table 1:** Patterns of smokeless tobacco use among women by type of user, and by specific product of use among single users.

Patterns of Smokeless Tobacco use	Sample percent (N = 409)	Types of smokeless tobacco						p value^^^
		Single Users (N = 261)					Poly Users^#^ N = 148%	
		*Mishri* N = 79%	*Pan* with tobacco N = 84%	Chewed Tobacco N = 29%	*Gu*l N = 43%	*Gutkha* N = 26%		
**Type of user**								
Single	63.8							
Poly	36.2							
**Frequency in a day (times)**								<0.001
1–2	25.7	38.0	42.9	3.4	46.5	34.6	6.1	
3–4	34.0	44.3	38.1	41.4	37.2	26.9	25.0	
5 and above	40.3	17.7	19.0	55.3	16.3	38.5	68.9	
**Amount of tobacco consumed in a day (grams)^1^**								<0.001
<5 gms	47.3	30.3	100.00	44.8	41.9	34.6	30.4	
> = 5 gms	52.7	69.7		55.2	58.1	65.4	69.6	
**Tobacco kept in mouth**								<0.001
<5 min	25.4	32.9	14.3	41.4	67.4	19.2	13.5	
5–10 min	39.4	26.6	48.8	27.6	25.6	34.6	48.0	
11–15 min	20.5	19.0	21.4	24.1	4.7	19.2	25.0	
>15 min	14.7	21.5	15.4	6.8	2.3	26.9	13.5	
**Use of tobacco after getting up in morning**								<0.001
≤ 5 min	18.3	44.3	1.2	48.3	51.2	11.5	0.0	
6–30 min	17.1	20.3	8.3	10.3	20.9	11.5	21.6	
31–60 min	27.9	7.6	16.7	13.8	9.3	11.5	56.1	
> 60 min	36.7	27.8	73.8	27.6	18.6	65.4	22.3	
**Product brought from market by**								<0.001
Self	64.3	82.9	58.3	72.4	62.8	84.6	52.7	
Children	22.0	11.8	20.2	10.3	14.0	7.7	35.8	
Other family members including husband	13.7	5.2	21.4	17.2	23.3	7.7	11.5	
**Native place**								<0.001
Maharashtra	19.8	75.9	2.4	10.3	2.3	19.2	6.8	
Uttar Pradesh	70.9	21.5	95.2	65.5	83.7	57.7	83.1	
Other State	9.3	2.5	2.4	24.1	14.0	23.1	10.1	

^#^ The distribution of SLT use among poly users—*Mishri* (35/148 = 24%), *Pan* with tobacco (112/148 = 76%), Chewed tobacco (75/148 = 51%), *Gul* (65/148 = 44%), G*utkha* (50/148 = 34%)

^ Chi-sq test for association between independent variable and the type of user (single/poly user)

Overall, the majority of women (75%) used each type of tobacco 3 or more times a day. However there were a few notable differences in the frequency of daily use of different types of tobaccos. Among single users approximately two-fifths of *mishri*, *pan* with tobacco and *gul* users rubbed 1–2 times a day. In contrast, more than two-thirds of poly SLT users (69%) used tobacco 5 or more times a day. Mean frequency of use among poly SLT users is approximately twice that of single users (7.6 vs 3.6; p<0.001).

The mean amount of SLT consumed by type of users (single vs poly) according to the specific product use show significant differences (Table 2). Among single users, the mean amount of SLT use per day by *mishri* users was 10.3 grams (± 9.0); by *gutkha* users was 11.9 grams (± 17.7); by chewed tobacco users was 7 grams (±5.0), by *gul* users was 6.9 grams (± 4.3) and by *pan* with tobacco users was 1.0 grams (±0.7). Among poly SLT users, the mean amount of SLT use per day by those who use *mishri* was 11.3 grams (±10.1); by those using *gutkha* was 14.3 grams (±10.5); by those using chewed tobacco was 11.8 grams (±8.5); by those who use *gul* was 8.9 grams (±6.2) and by those using *pan* with tobacco was 8.4 (±7.6). Overall, poly SLT users chewed or rubbed 50% more grams of tobacco compared to single users (mean consumption of tobacco per day: 9.54 vs. 6.49 grams; p<0.001).

**Table 2:** Mean tobacco consumption (amount in grams per day) by single and poly users according to the specific product used.

Users of specific product	Amount of tobacco consumption per day (in grams)		p value
	Single user N = 261 Mean± SD	Poly user* N = 148 Mean± SD	
*Mishri*	10.3±9.0	11.3±10.1	0.628
*Pan* with tobacco	1.0±0.7	8.4±7.6	<.001
Chewed Tobacco	7.5±5.0	11.3±8.5	0.028
*Gul*	6.9±4.3	8.9±6.2	0.054
*Gutkha*	11.9±17.7	14.3±10.5	0.484
Overall	6.5±8.8	9.5±8.2	0.001

* Poly users used the specific product in combination with other products in a day. Mean tobacco consumption refers to the total amount of tobacco consumed from all the products among those who use that specific product.

Most women, (more than 75%) reported keeping tobacco inside the mouth for more than 5 minutes, although duration varies across tobacco types (Table 1); the percentage of women keeping tobacco inside the mouth for more than five minutes is somewhat higher among poly SLT users (87%) than among single users (68%) (p<0.001).

Timing of first use every day varies by product as well as user. Among single users, 60–70% of *mishri*, tobacco and *gul* users chewed or rubbed tobacco within the first 30 minutes of awakening. Approximately 70% of *gutkha* and poly SLT users started their use later, from 30–60 minutes after getting up; *pan* with tobacco chewers also chewed their first *pan* over an hour after getting up.

The quotes below reflect examples of the way women schedule their use of different forms of tobacco throughout the day in accordance with their daily hygiene, work, meals and child care rituals.

“Madam after every 1 and ½ hour, I was eating *gutkha* till night. And when I was not eating *gutkha* at that time I would eat *pan*. See after eating *gutkha* I keep that in the mouth for half an hour. Then I spit, but *supari* (betel nut) I take it inside——-—I keep it for 10 to 15 minutes. In *pan* also I like the *supari*, so I do the same thing. The water I spit, supari I eat. *Pan* and *guthka* are different; it does not have the same taste; *pan* is spicy and it gives “*jum jum*” feeling in the teeth. But in *gutkha* there is less spice and the smell is good. It gives freshness in the mouth—”. {*PD 10*: *22 year old pregnant woman*, *poly SLT user (pan with tobacco*, *gutkha and gul)*, *one child}*

“When I wake up I rub *mishri* and go to the toilet, I keep it in mouth for 20 minutes. After coming back I clean my mouth and again brush my teeth with Colgate because the particles of *mishri* remain inside the teeth. At 11:00 a.m. I eat tobacco and after fifteen minutes spit that. After drinking tea I sit to wash utensils and clothes. Then in between I again eat tobacco. Till 1:00 p.m. my children come for lunch so we sit together and have lunch. After lunch I eat tobacco and after cleaning my mouth I go to sleep from 2:00 p.m. to 4:30 p.m. But it depends suppose. If I am not sleeping for a long time then I eat tobacco at 3:30 p.m., otherwise I don’t. Before going to the market at 5:00 p.m. in the evening I eat tobacco and then I rinse my mouth. Till today, I have never eaten tobacco on the road and before going outside I don’t like to eat. When I come back then I eat tobacco till 7:30 p.m. When my daughter prepares food I sit outside with my neighbour and chat, and that time I eat *gutkha* with all of them. At 10:00 p.m. my husband comes back from work so we have dinner together. And after having dinner I eat tobacco and I spit and go to sleep”. *{PD 26*: *32 year old*, *poly SLT user (tobacco*, *mishri and gutkha) with three children}*.

The majority of women purchase their SLT products by themselves from *pan* shops or general stores when they are shopping for other goods (Table 1). Women also report that their husband or other family members bring *pan* with tobacco for them to eat together or purchase tobacco for them.

“I go myself and buy it very few times; mostly my husband brings it for me.” *{PD 16*: *25 year old pregnant woman*, *single user (mishri) with two children}*

Women say that children purchase all types of tobacco. While single users report buying products on their own, a relatively higher percentage of poly SLT users reported that children bought products for them.

“Sometimes I tell my children to purchase 10 to 15 packets at a time. I hide it and keep”. {*PD 21*: *21 year old poly SLT user (tobacco*, *gutkha and mishri) with one child}*.

**“I:** From where do you purchase? Is there any particular shop? **P:** Yes on the main road there are two—three shops, from there my children bring. **I:** What type of order do you give your children if they have to bring *pan* for you? **P:** My children know what type of *pan* I eat. So they bring *pan* with *surti* tobacco and ripe betel nut (*pakki supari*) {*P33*: *30 year old pregnant woman*, *single user (pan with tobacco) with four children}*

Seventy-one percent of respondents were from Uttar Pradesh (U.P); 20% were from Maharashtra and the remainder were from other areas of India. *Mishri* was reported primarily by women from Maharashtra, but some were also from U.P. Most poly SLT users were from the state of Uttar Pradesh but approximately 30% of m*ishri users* were also poly SLT users.

Among users of all types of tobaccos there were few significant differences by background characteristics. Generally women from Maharashtra were more likely to use m*ishri* whereas women from Uttar Pradesh and other states were more likely to use *pan* with tobacco, *gutkha*, *gul* and chewed tobacco. *Gutkha* users were more likely to be younger in age. There were no significant differences in type of tobacco used by pregnancy status, the husband’s use of SLT, duration of stay in Mumbai, type of family, family income, or childhood exposure to the product.

### Sequence of Initiation of Tobacco Types

Among women poly SLT users, the progression of use of different types of tobacco takes several different forms. As indicated, the study included 148 poly SLT users (109 using 2 products, 33 currently using three products, and 6 using four products). Table 3 considers only first degree of progression of SLT use, i.e. from the first to the second product, and shows 10 different combinations of poly SLT users. Most of the women initiated with *pan* with tobacco (41), followed by chewed tobacco (28), *gutkha* (17), *mishri* (15) and *gul* (14). Among poly SLT using *mishri* users, 15 initiated with *mishri* and started using *pan* with tobacco, *gutkha* and chewed tobacco, whereas very few initiated with *pan* with tobacco (5) or chewed tobacco (3). Twenty-two percent initiated tobacco use with two forms of tobacco. Twenty-eight percent added a second form of tobacco within 2 years after the first use, and the remainder added tobaccos over a period of approximately five years.

**Table 3:** Order in which poly smokeless tobacco users started using different products.

Type of smokeless tobacco		Poly smokeless tobacco users who reported initiating			Total
A	B	A first N	B first N	Same time* N	
*Mishri*	*Pan* with tobacco	9	5	4	18
*Mishri*	*Gutkha*	3	0	3	6
*Mishri*	Chewed tobacco	3	3	1	7
*Mishri*	*Gul*	0	0	1	1
*Pan* with tobacco	*Gutkha*	4	7	3	14
*Pan* with tobacco	Chewed tobacco	15	13	9	37
*Pan* with tobacco	*Gul*	17	9	10	36
*Gutkha*	Chewed tobacco	6	5	0	11
*Gutkha*	*Gul*	4	1	1	6
Chewed tobacco	*Gul*	7	4	1	12
**Total**		**68**	**47**	**33**	**148**

*Women who initiated the use of two smokeless tobacco products in the same year/age

The qualitative data offers some clues as to patterns of increase or decrease over time. For example, women tend to add a new product to their repertoire when they want more “kick”. They may add or substitute a new tobacco when they want to reduce their intake of their currently used tobacco. Switching or supplementing is common when the cost of the tobacco of preference rises or when they can no longer procure a favorite type of tobacco because it is unavailable, or banned. Women may initiate or switch products during pregnancy because they like or dislike the smell or taste, or they may stop using SLT altogether. Cost also affects choice and amount of use; an increase in cost of one type of tobacco may result in a switch to cheaper brands or types of tobacco.

Following is an example of a woman who had increased and then decreased her use of *gutkha* during pregnancy.

“Initially I used to have 2–3 times in a day, at times 3–4 times in a day and then one packet in a day, then 2 packets in a day. I eat only *gutkha*, because I like the taste. It contains *supari* (betel nut), chemicals, *zarda* and tobacco. When I came to know that I am pregnant that time I felt more craving and started eating 15–20 packets in a day. But my sister, mother-in-law and husband made me understand that it is not good for me because I am pregnant. Then I reduced eating and started eating only 4 times in a day”. {*PD15*: *19 year old*, *1* ^*st*^ *pregnancy*, *single user (gutkha)}*

### Smokeless Tobacco Use within Social Networks

One factor contributing to SLT use is the use of brands and types of tobacco in close personal networks. Overall totals for the number of types of tobaccos participants listed as used by members in all five of their close personal social networks (non-spousal household members, relatives in Mumbai, relatives outside of Mumbai, friends and neighbors) ranged from 0–20 with a mean of 6±3.2. The mean overall differences between single and poly users in exposure to number of tobacco types across personal networks were significant (p<.001) (Table 4). Mean exposure to types of tobacco was significantly higher among poly SLT users than single users for relatives in Mumbai (p<.001), friends (p<.02), and neighbors (p<.001), but not for relatives outside of Mumbai or other non-spousal household members.

**Table 4:** Mean difference in exposure to number of types of tobacco by different components of personal network and type of smokeless tobacco user.

Exposure to types of tobacco in personal network	Type of users		p value^$^
	Single users N = 261 Mean ±SD	Poly user N = 148 Mean ±SD	
**Social network components**			
Relatives in Mumbai	1.57±1.18	2.05±2.23	<0.001
Neighbors	1.65±1.24	2.14±1.38	<0.001
Friends	0.59±0.84	0.83±1.18	0.022
Non-spousal household members	0.43±0.74	0.37±1.20	0.249
Relatives outside of Mumbai	1.62±1.05	1.82±1.31	0.111
Overall	5.8 ± 2.6	7.3 ±3.6	<0.001

* Individual score ranges from 0–5 and overall score ranges from 0–25

^$^ t-test for difference between means

### Differences in Tobacco Use between Pregnant and Non-Pregnant Women

There were no significant differences between currently pregnant and non-pregnant women by type of users (single vs poly SLT user), or daily frequency or amount of use. Further, though the survey data revealed no consistent trajectories of change in SLT use across pregnancies, the following qualitative examples show that there are increases and decreases in SLT use as well as consistency of use prior to and during pregnancy.

In the following example, a woman describes her continuing use of SLT during pregnancy and up to the present.

“I started having *pan* and raw tobacco before my marriage. In my family everyone eats *pan* and tobacco. When I was pregnant for the first time my husband came to know. He scolded me however by then I was habituated. I continued using even in pregnancy and I have it up till now”. {*PD 20*: *30 year old pregnant woman*, *poly SLT user (pan with tobacco and chewed tobacco) with two children}*.

The next example describes a woman who continued to use SLT at the same level when she found out that she was pregnant.

“The quantity of tobacco during pregnancy was the same like before. I didn’t tell the doctor though I have some idea that it is injurious to health. But till date I never feel it is dangerous to health because I have never faced any health problem. Or whenever I feel like eating I eat tobacco. Without tobacco I can’t live. Madam I will tell you one story of my life. At the time of my first delivery, when the ward boy shifted me to the bed and left, I opened the packet and made tobacco and put it in my gum. Then I felt relief because during delivery when I was having labour pain that time I was very tired so I felt like eating tobacco…” {*PD 24*: *She is a 32 year old*, *poly SLT user (chewed tobacco*, *mishri) with two children}*.

The example below illustrates how a woman added a new type of SLT to her repertoire during pregnancy.

She says “…in the second pregnancy, my mother-in-law was telling me to eat three *pan*; it doesn’t matter if I have gas problem or not. I don’t know the reason for suggesting me to eat. I have never increased my intake even in the third pregnancy; I only ate three *pans* since the beginning. Sometimes I use tobacco. This I started now in my present pregnancy because I had a craving to eat something different. So I thought of using this tobacco. No one introduced me to this tobacco. I only brought it from the shop because now I have some kind of vomiting sensation. So I tried this. When I don’t eat *pan* that time I eat tobacco mixed with lime”. *{PD 37- She is 35 year old pregnant woman*, *poly SLT user (pan with tobacco and chewed tobacco) with four children}*.

The next quote describes how and why a woman reduced or quit her use of SLT during pregnancy.

“Madam as soon as I came to know that I was pregnant I had completely stopped eating *pan* and *gutkha* because I hated the smell and because I had vomiting. So for complete 9 months I had stopped eating *pan* and gutkha, because of vomiting my mouth would become very dry and I would never feel the taste in anything.” {*PD44*: *She is a 30 year old*, *poly SLT user (pan with tobacco*, *gutkha) with 4 children}*.

### Predictors of Poly Smokeless Tobacco Use

To identify socio-demographic and contextual factors associated with poly SLT use, a multiple logistic regression analysis was conducted (Table 5).

**Table 5:** Factors associated with poly smokeless tobacco use by selected background characteristics among women in Mumbai.

Background Characteristics	Type of user		Unadjusted Odds Ratio UOR (95% CI)	Adjusted Odds Ratio AOR (95% CI)
	Single user (N = 261) %	Poly user (N = 148) %		
**Age**				
≤30 yrs	52.9	53.4	1.00	1.00
>30	47.1	46.6	0.98 (.65–1.47)	0.89 (0.54–1.46)
**Currently pregnant**				
No	75.5	74.5	1.00	1.00
Yes	24.5	25.7	1.06 (0.67–1.69)	1.16 (0.68–1.96)
**Education**				
Literate	50.2	37.2	1.00	1.00
Illiterate	49.8	62.8	1.70 (1.13–2.57)	**1.67 (1.07–2.60)**
**Duration of stay in Mumbai**				
0–10 yrs	41.0	35.1	1.00	1.00
>10 yrs	59.0	64.9	1.28 (0.84–1.95)	**1.67(1.03–2.71)**
**Type of family**				
Nuclear	72.0	77.7	1.00	1.00
Joint	28.0	27.6	0.74 (0.46–1.18)	0.83 (0.49–1.37)
**Family income**				
<5000	26.1	30.4	1.00	1.00
≥5000	73.9	69.6	0.81 (0.52–1.26)	0.94 (0.57–1.5)
**Childhood exposure to any product**				
No	18.4	16.2	1.00	1.00
Yes	81.6	83.8	1.16 (0.68–1.99)	1.13 (0.63–2.0)
**Husband’s use of SLT**				
Non user	31.8	21.6	1.00	1.00
Single	39.8	24.3	0.89 (0.52–1.57)	0.86 (0.49–1.53)
Poly SLT user	28.4	54.1	2.80 (1.67–4.69)	**2.78 (1.63–4.76)**
**Tension**				
No	47.5	40.5	1.00	1.00
Yes	52.5	59.5	1.32 (0.88–1.99)	1.22 (0.78–1.89)
**Food insufficiency**				
No	36.0	25.7	1.00	1.00
Yes	64.0	74.3	1.63 (1.04–2.55)	1.49 (0.93–2.41)

After controlling for several background characteristics, literacy, number of years of stay in Mumbai and husband’s use of SLT were found to be associated with poly SLT use. Illiterate women users were almost two times more likely to be poly SLT users than literate users (Adjusted Odds Ratio [AOR] = 1.67, 95% confidence interval [CI] = 1.07–2.60). Women who had lived in Mumbai 10 years or more were almost two times more likely to be poly SLT users than their counterparts (AOR = 1.67, 95% CI = 1.03–2.71). Women, whose husbands were poly SLT users, were three times more likely to be poly SLT users than those whose husbands were non-users (AOR = 2.8, 95% CI = 1.63–4.76). Being pregnant was not associated with either single or poly SLT use.

## Discussion

In this study, we examined the tobacco use patterns of married women of reproductive age (18 to 40 years) who were daily users of at least one SLT product. No women in our study reported smoking cigarettes; this is consistent with the 2010 Global Adult Tobacco Survey (GATS) data showing that only 2 of 1219 urban Maharashtrian women smoked at all. The estimated prevalence of SLT use in this population of urban, low income, married women was 31.5%, the highest on record for urban women in Western India. Of these, more than one third reported use of more than one SLT daily. In contrast, the GATS data for Maharashtrian urban women respondents shows that of women aged 16–40 years, 8.3% reported the use of 1 type of smokeless tobacco daily and an additional 1.4% reported less than daily SLT use. Of these only approximately 3% reported the use of two or more forms of SLT [2].

Women reported the use of five main types of smokeless tobacco: *pan* with tobacco, *mishri*, *gutkha*, chewed tobacco and *gul*. Other studies carried out in Mumbai [36] and other parts of Maharashtra report the predominance of *mishri* use among women in Mumbai and Maharashtra overall [37,38]. A study conducted in a maternity home in Delhi reported that most of the women used *gul* (66%), with *gutkha* and chewed tobacco as the next two favored substances; both typically used in northern India [39]. In our study, the majority of *mishri* users, 57.3%, were from Maharashtra, but 35.5% of *mishri* users were from U.P. and some Maharashtra-born *mishri* users also used *pan* with tobacco, chewed tobacco and *gutkha*. This mixed pattern of use in the study community is likely due to exposure to multiple types and brands of smokeless tobacco sold in shops, used by family members, and observed among neighbors and others in the community. In urban slum areas, the ready availability of many types of SLT used in different parts of the country offers women options as they switch or add different types of tobacco to their repertoires [31].

The average period of time required for full absorption of nicotine is twenty minutes, although timing varies with dose and condition; for example, absorption of powdered tobacco through the gums is more rapid [40–42]. The majority of m*ishri or gul users* keep it in their mouth for less than 5 minutes, while women using other forms of tobacco reported keeping it in the mouth for 5–20 minutes or more. Food insufficiency was not a statistically significant predictor of poly SLT use in this sample. Thus it appears that women keep tobacco in their mouth for as long as necessary to derive maximum absorption of nicotine in whatever substance they are using and are not chewing it as a substitute for food.

One measure of addiction is use of a tobacco product within 5 minutes of getting up in the morning [43,44]. Unlike cigarette smoking, timing of first use varies significantly across products and across SLT users. For example, half of all *mishri* users rub it on their teeth and gums within the first five minutes of awakening, while *gutkha* and *pan* with tobacco chewers use it for the first time later in the day and *gul* users can start their use at any point. *Mishri* is often used as a dentifrice and hence probably why it is used within the first five minutes of awakening. Thus the Fagerstrom measure of addiction is not useful in the Indian context, at least with respect to smokeless tobacco use. Other indicators such as frequency of use, urge to use, withdrawal symptoms [45,46] or poly SLT use are likely to be more useful in assessing addiction.

Though there is reported dual use of cigarettes and smokeless tobacco in India among men [47], there is little research as yet on poly SLT use among women. Understanding poly SLT use is particularly important because users of more than one type of tobacco often ingest more tobacco per day, with a significant dose response effect on reproductive as well as oral health outcomes. A study conducted in Mumbai showed that 18% of women used *mishri* or *pan* with tobacco and around 3.3 percent of women reported multiple practices (i.e. simultaneous use of *mishri* or *pan* with tobacco or *pan* or tobacco) [36]. Our study suggests that, two decades later, multiple use of SLT has become significantly more common. Over one third of our study population of daily SLT users (36.2%) used more than one type of tobacco daily, and some used three or even four different types of tobacco, rubbing or chewing each type of tobacco more than once or twice a day. For poly SLT users, quitting one form of tobacco may even lead to an increase in others, depending on availability and flavor. Just after the 2012 *gutkha* ban, follow-up interviews with *gutkha* users showed that most of them switched to and increased other forms of tobacco [32]. A comparison of frequency and gram consumption showed that overall, poly SLT users used SLT nearly twice as often as single users and ingested approximately one third more grams of tobacco.

Women’s tobacco use is closely intertwined with and timed in accordance with their daily activities. Women start the use of tobacco at different times of the day, and describe how they organize their use in accordance with their daily activities, e.g. starting the day with *mishri* use, organizing *mishri* rubbing or chewing around periods of intensive work during morning or afternoon, chewing *pan* with tobacco at night. This pattern of integrating tobacco use with work, socialization and relaxation has the potential for increasing the difficulty of quitting the use of tobacco. At the same time, it offers the opportunity to measure stages of addiction by identifying “atypical” patterns of use for each type of SLT, such as *mishri* use throughout the day rather than morning and evening for teeth cleaning.

A high percentage of women are surrounded by others who chew or rub tobacco, including their husbands, other members of their households, relatives, friends and neighbors. Study results showed that the use of more types of tobacco in close personal networks is associated with poly SLT use. The analysis also showed that poly SLT use is associated with more types of tobaccos used by relatives staying in Mumbai, friends and neighbors but not with relatives outside of Mumbai, suggesting that proximity of users is important in creating a pro-tobacco environment and influence to use.

Husbands are an especially important influence in women’s SLT use. Husbands bring their wives tobacco products, share tobacco use with them, and store their chewed tobacco and *gutkha* at home. Thus women can easily access the stronger brands of chewed tobacco and *gutkha* (or *gutkha*-like products) that men prefer. Women whose husbands are poly SLT users are much more likely to be poly SLT users themselves. In addition to obtaining tobacco from their husbands, most women purchase their own tobacco and some send their children to obtain a supply for them. Social norms also endorse or at least condone women’s use of SLT [31] making it easy for women to obtain and to use tobacco in public. Though tobacco control regulations prohibit the sale of tobacco to children, social norms support it. By purchasing tobacco for their mothers, children are exposed to SLT use at point of sale as well as at home, and to the possibility of early initiation of SLT products [48,49].

Our study shows that frequency and amount of SLT use does not vary with pregnancy status, suggesting that women do not change their use of tobacco during pregnancy. Almost all women in both the qualitative and the survey sample reported continuing their use of SLT during pregnancy. Earlier studies in India also reported women’s use of tobacco during pregnancy [50]. In addition, our research has shown that initiation of tobacco use often occurs during pregnancy because of the belief that tobacco counters the weakening of teeth and gums that occurs during pregnancy. The in-depth interviews conducted in our study did provide several examples of women who increased, decreased or even stopped the use of one or more SLT products during pregnancy, but decreasing or quitting use did not persist post-birth. Similar findings are reported among pregnant Alaska Natives using SLT [51]. In India the few studies that describe SLT intervention efforts during pregnancy show limited influence on reduction of SLT use during pregnancy and no influence on either reduction or cessation post pregnancy [37]. Thus in addition to viewing pregnancy as an opportunity for tobacco control and prevention as suggested in recent publications [52,53], debunking myths about the benefits of tobacco use prior to pregnancy could be equally valuable in the India context.

The study results may be considered in light of certain limitations. The study is based in a single slum community of 65,000 people and thus, may not be generalizable. The calculations of gram usage were based on the weight of product by product unit (packet, teaspoon, etc.), and self-reported frequency and amount of usage, but may not be fully accurate. Further, it was not possible to determine exact amount of nicotine in the tobacco products. The study was designed to examine patterns of use among pregnant and non-pregnant SLT users, not predictors of initiation; thus it identified risk factors for poly SLT use but could not compare users and nonusers.

## Conclusion

Smokeless tobacco is inexpensive, readily accessible, and unlike smoked tobacco, its use among women is widely accepted and acceptable [31]. Once women become daily users, they maintain or increase their level of tobacco consumption either by increasing amount of use, adding products, or both. This occurs regardless of whether they are pregnant or not. The use of any SLT is considered to be hazardous, and daily users of one type of tobacco may use as much as some poly users. But this study shows that poly SLT users use smokeless tobacco more frequently and use more than single tobacco users. Thus, they represent a special challenge for regulatory action such as bans, or prevention and cessation programs, since poly SLT users may shift more easily from one type of tobacco to another and it may be more difficult for women to quit the use of more than one type of tobacco. The ease with which women can access, purchase and use SLT without stigma, the fact that they are surrounded with users, and the way it is embedded in the routines of daily life all increase the likelihood of using one or more tobacco products.

Single approaches to reducing SLT among women are unlikely to be successful given the complexity of the SLT problem at the community level. Needed are comprehensive programs that: 1) reduce the ease with which women obtain tobacco by purchasing it themselves or asking others to buy it for them; 2) engage husbands and other close personal family and friends in anti-tobacco efforts; 3) promote anti-tobacco norms; 4) reinforce regulations preventing the sale of tobacco to children; 5) offer opportunities for treatment prior to as well as during pregnancy; and 6) regulate and stigmatize public use of all forms of SLT.
